# X-ray Spectroscopy Study of Defect Contribution to Lithium Adsorption on Porous Carbon

**DOI:** 10.3390/nano13192623

**Published:** 2023-09-22

**Authors:** Yuliya V. Fedoseeva, Elena V. Shlyakhova, Anna A. Makarova, Alexander V. Okotrub, Lyubov G. Bulusheva

**Affiliations:** 1 Nikolaev Institute of Inorganic Chemistry, Siberian Branch of Russian Academy of Sciences, 3 Acad. Lavrentiev Ave., Novosibirsk 630090, Russia; shlyakhovaev@niic.sbras.ru (E.V.S.); spectrum@niic.nsc.ru (A.V.O.); 2 Physikalische Chemie, Institut für Chemie und Biochemie, Freie Universität Berlin, 14195 Berlin, Germany; anna.makarova@fu-berlin.de

**Keywords:** porous carbon, lithium adsorption, NEXAFS, XPS, DFT, lithium-ion batteries

## Abstract

Lithium adsorption on high-surface-area porous carbon (PC) nanomaterials provides superior electrochemical energy storage performance dominated by capacitive behavior. In this study, we demonstrate the influence of structural defects in the graphene lattice on the bonding character of adsorbed lithium. Thermally evaporated lithium was deposited in vacuum on the surface of as-grown graphene-like PC and PC annealed at 400 °C. Changes in the electronic states of carbon were studied experimentally using surface-sensitive X-ray photoelectron spectroscopy and near-edge X-ray absorption fine structure (NEXAFS) spectroscopy. NEXAFS data in combination with density functional theory calculations revealed the dative interactions between lithium sp^2^ hybridized states and carbon π*-type orbitals. Corrugated defective layers of graphene provide lithium with new bonding configurations, shorter distances, and stronger orbital overlapping, resulting in significant charge transfer between carbon and lithium. PC annealing heals defects, and as a result, the amount of lithium on the surface decreases. This conclusion was supported by electrochemical studies of as-grown and annealed PC in lithium-ion batteries. The former nanomaterial showed higher capacity values at all applied current densities. The results demonstrate that the lithium storage in carbon-based electrodes can be improved by introducing defects into the graphene layers.

## 1. Introduction

Lithium-ion batteries (LIBs) are widely used in portable electronics due to their high energy density, light weight, safety, and stable operation. Graphite, the main commercial anode material in LIBs, stores Li in its interlayer space at a low operation potential of 0.1 V vs. Li/Li^+^. The limiting content of lithium is one Li per six carbon atoms, which ensures a relatively low theoretical capacity of 372 mAh g^−1^ [1,2]. To increase the capacity of anode materials, it is necessary to maximize the amount of accumulated lithium and charge transfer to the carbon network. The porous structure of carbon nanomaterials allows more lithium to be stored and ensures fairly rapid mass transfer. Porous carbon (PC) nanomaterials significantly enhance the power and long-term operation of LIBs and can be used to manufacture flexible electrodes [3,4,5,6]. Thin, few-layered graphitic materials with high surface-to-volume ratios demonstrated high storage capacity as the anode in LIBs ranging from 370 to 1054 mAh g^−1^ [7].

According to the data from cyclic voltammetry (CV) and in situ Raman spectroscopy, lithium accumulation on the surface of a defect-free graphene monolayer is significantly less than in fully lithiated graphite LiC_6_ and multilayer graphene owing to strong repulsion between adsorbed Li^+^ ions in the former case [8]. The binding energy of lithium to graphene is lower than that of graphite but higher than that of metallic lithium clusters [9]. At high concentrations, Li atoms aggregate into small clusters on the graphene surface, which can lead to poor anode stability due to the formation of Li dendrites. In contrast to defect-free graphene, numerous 3D graphene nanomaterials composed of corrugated layers exhibit outstanding electrochemical Li storage performance, high gravimetric capacity and rate capability, and stable long-term cycling, which is attributed to the porous structure and high defect density [10].

The lithium storage capacity of amorphous carbon and 3D graphene anodes is two to three times higher than that of a graphite anode and is maintained even at high charge-discharge rates [11,12,13,14,15,16]. For example, interconnected carbon nanoflakes with a hierarchical porous structure showed the capacity of 802.6 mAhg^−1^ at a current density of 0.5 Ag^−1^ [16], while the capacity of 3D graphene nanomaterial reached 1000 mAh g^−1^ at 4 Ag^−1^ [12]. The advantage of PC nanomaterials is their simple and low-cost synthesis, such as carbonization of biomass, coal, oil, organic compounds, carbon-containing gases, etc. [17,18,19]. Template nanoparticles are used in the carbonization process to increase and control the porosity of nanomaterials [20,21]. Previously, we developed a method for synthesizing a carbon material consisting of graphene containing a few layers and enriched with interconnected mesopores using chemical vapor deposition (CVD) of ethanol or another carbon source onto CaO template nanoparticles produced by pyrolysis of calcium tartrate [22]. This allows us to control the pore size and specific surface area by changing the synthesis temperature and composition of the template and carbon precursors. The resulting carbon materials demonstrate excellent characteristics in supercapacitors and lithium- and sodium-ion batteries [23,24,25].

Nuclear magnetic resonance (NMR) spectroscopy studies revealed that Li can coexist in porous carbon nanomaterials in two forms: intercalated between graphene layers and adsorbed on their surface [26]. Surface adsorption of Li occurs in a wide potential range from 0.1 to 1.5 V vs. Li/Li^+^ and is the main mechanism for storing Li in porous carbon. Therefore, the amount of electrochemically adsorbed Li is affected by the carbon structure, namely, the thickness and lateral size of graphene layers, surface accessibility, defect states, functional groups, and electrical contact between nanoparticles. There is still no unambiguous understanding of the role of structural defects in the adsorption and chemical interaction of lithium with carbon.

The chemical bonding between Li and graphene, as well as with metal oxides, is generally considered to be predominantly ionic with strong charge transfer from Li to C or O atoms [27,28]. However, the covalent contribution to the Li–C bond is not negligible. Mederious et al. used quantum chemical calculations to study the bonding of Li atoms to a graphene layer and revealed the interaction between p_z_ orbitals of carbon atoms and Li sp^2^-hybridized orbitals, which corresponds to the mixed covalent–ionic character of Li–C bonds [29,30]. The contribution of the Li 2p orbitals to these bonds is the reason why Li is located over the center of the carbon hexagon rather than over the C atoms, as is the case for hydrogen. Although the effect of defects on Li adsorption is well described theoretically from an energetic point of view, there are few works that experimentally examine the chemical bonding between lithium and defective graphene nanomaterials. The main experimental challenges for this are the disordered structure and instability of lithiated carbon in air, the presence of surface impurities from the electrolyte, and the limitation and complexity of the characterization tools. To overcome these problems, in situ investigations of lithiated carbon are usually carried out using microscopy [31], Raman spectroscopy [32,33], and NMR spectroscopy [34]. Soft X-ray spectroscopy methods provide detailed information about the local chemical environment of atoms and densities of electronic states. In situ studies of carbon nanomaterials by X-ray photoelectron spectroscopy (XPS) and near-edge X-ray absorption fine structure (NEXAFS) spectroscopy using synchrotron radiation revealed changes in the electronic structure of carbon materials after their interactions with alkali metals in vacuum [25,35,36,37].

Here, we focus on the role of native vacancy defects in the graphitic structure of PC in Li accumulation. Small vacancies, in which one or two neighboring carbon atoms are missing, inevitably form during the growth of PC and its purification by acids. It was shown theoretically that single-vacancy or double-vacancy defects can increase the Li storage capacity of graphene [38,39]. In this work, we carried out a comparative study of the ability of PC nanomaterial, as-synthesized and annealed at 400 °C in vacuum, to accumulate thermally evaporated lithium atoms. We used XPS and NEXAFS spectroscopy tools to study the surface composition and electronic structure of lithiated samples. Quantum chemical modeling within density functional theory (DFT) was used to explain a new feature in the NEXAFS C K-edge spectra of lithiated samples. It was found that atomic vacancy defects in the graphene structure not only enhance the binding energy of the primary attached Li atoms but also provide more acceptor states, increasing the electron density transfer from Li atoms and the ionic character of carbon–lithium bonding. This study sheds light on the vital role of defects in the Li-ion storage behavior of carbon anodes in LIBs. This issue is also relevant because carbon materials are widely used as an active component of composite electrodes based on silicon and metal oxides.

## 2. Materials and Methods

### 2.1. Synthesis

**Figure 1:**
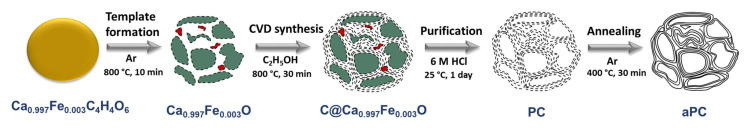
Scheme of the synthesis procedure for PC and aPC samples. The green and red areas correspond to calcium oxide and iron oxide, respectively, while the gray lines indicate graphene-like layers.

PC was synthesized by ethanol CVD on a calcium oxide template. Figure 1 schematically shows the synthesis procedure. Synthesis details are described in [23]. Template nanoparticles were obtained by pyrolysis of calcium tartrate doped with iron (0.4 wt%) in an argon atmosphere at 800 °C for 10 min. After that, ethanol vapor was injected into the reactor at the same temperature for 30 min by passing an argon flow through liquid ethanol heated to a temperature of 35 °C. The sample was cooled inside the reactor in an argon atmosphere to room temperature. The resulting product was purified from template nanoparticles using a 6 M aqueous hydrochloric acid solution for 1 day. The purified PC material was repeatedly washed with distilled water to a neutral pH value and dried at 100 °C for 2 h. Annealing of PC was carried out in a quartz tube filled with argon at a temperature of 400 °C for 30 min. This annealed aPC sample was used in the LIB studies.

### 2.2. Characterization

Scanning electron microscopy (SEM) images were obtained on a Hitachi S-3400N microscope (Hitachi Ltd., Tokyo, Japan) at an accelerating voltage of 5 kV in secondary electron imaging mode. Transmission electron microscopy (TEM) images were obtained on a JEOL 2010 microscope (JEOL Ltd., Tokyo, Japan) with an accelerating voltage of 200 kV. Raman spectra were measured on a LabRAM HR Evolution instrument (Horiba, Kyoto, Japan) using a He–Ne laser with a wavelength of 633 nm.

X-ray emission spectroscopy (XES) experiments were carried out on a “Stearat” laboratory X-ray spectrometer. An ammonium biphthalate single crystal was used as an analyzer crystal to obtain the C K_α_ spectra. The natural flake crystalline graphite (GT-1 brand according to GOST 17022) purified in a mixture of hydrochloric and nitric acids to a metal content of 10^−2^ wt% was used as a graphite sample. To prevent degradation of the samples under the action of X-rays, they were cooled to the temperature of liquid nitrogen. During the measurement, the residual pressure in the chambers of the spectrometer was ~10^−6^ Torr.

XPS and NEXAFS measurements were performed at the experimental station of the Russian–German beamline at the Berlin Helmholtz Centre for Materials and Energy (HZB, Berlin, BESSY II). The XPS spectra were collected using a Phoibos 150 hemispherical analyzer and a monochromatic photon beam of energy at 830 eV as an excitation source. The energy of the spectral components was calibrated against the Au 4f_7/2_ line at 84.0 eV and measured from a cleaned gold foil. The NEXAFS spectra were recorded in the total electron yield mode. PC samples were applied to two rough copper substrates, which were fixed on two different holders and placed in a preparation chamber evacuated to an ultrahigh vacuum (UHV) of ~10^−10^ Torr. One of the substrates fixed on a holder with a tantalum wire was annealed at a temperature of 400 °C for 20 min in the UHV preparation chamber. The annealed sample is designated as aPC. The deposition of Li was performed equally on the surfaces of PC and aPC in the UHV preparation chamber by applying direct current (7.5 A) to a preliminarily degassed Li source (SAES Getters) for 25 min. The initial and annealed samples after lithium deposition are designated as Li + PC and Li + aPC. Immediately after lithium deposition, the samples were transferred to the analytical chamber, where the XPS and NEXAFS spectra were recorded at the same parameters as for the initial samples.

### 2.3. Electrochemical Measurements

Electrochemical tests of the PC anodes in LIBs were carried out in a two-electrode configuration on a NEWARECT-3008 charge–discharge station (Neware Techonology Ltd., Shenzhen, China) at room temperature. The measurements were performed in the galvanostatic mode in the voltage range from 2.50 to 0.01 V vs. Li/Li^+^ at current densities of 50, 100, 250, 500, and 1000 A g^−1^. The CV curves were obtained using a Bio-Logic SP-300 potentiostat–galvanostat (Bio-Logic Science Instrument, Seyssinet-Pariset, France) in the voltage range from 2.50 to 0.01 V vs. Li/Li^+^ at a potential scan rate of 0.5 mV s^−1^. For the fabrication of working electrodes, PC and aPC powders were ground in a mortar and mixed with a polyvinylidene fluoride binder and N-methyl-2-pyrrolididone solvent (taken in a mass ratio of 8:1:1). To achieve better adhesion of carbon materials to the substrate and their economical consumption, Ni foam was used as a current collector. Nickel foam disks were uniformly soaked with the resulting slurries, dried at 100 °C for 10 h, and then pressed. The mass of the dried working electrodes was about 2 mg. A Li metal sheet, polypropylene separator, and electrolyte (1 M LiPF_6_ solution in equal volumes of ethylene carbonate and dimethyl carbonate) were packaged with the working electrode in CR2032 coin-type cells inside an argon-filled glove box.

### 2.4. Calculation Details

To explain the changes in the shape of NEXAFS C K-edge spectra as a result of Li deposition, we analyzed the partial densities of unoccupied states for three models of graphene: a defect-free fragment (G, C_80_H_22_) and fragments with single vacancy (SV, C_79_H_22_) and diatomic vacancy (DV, C_78_H_22_). DFT calculations were carried out using the M06 hybrid functional parametrized for metal-containing systems in order to describe organometallic compounds and noncovalent interactions [40] within the Jaguar software package (version 10.3, Schrödinger, Inc., New York, NY, USA, 2019) [41]. Atomic orbitals (AO) were described by the 6–31 g basis set. The geometry of the models was optimized by the analytical method up to a gradient of 5 × 10^−4^ atomic units to shift the positions of all atoms. Li atoms were successively added to one side of the graphene models G, SV, and DV. The geometry of lithiated models was optimized under the same conditions as initial models. Below, the lithiated models are designated nLi-G, nLi-SV, and nLi-DV, where n is the number of Li atoms. The adsorption energy was calculated for each extra Li atom as the difference between the total energy of the nLi-G, nLi-SV, or nLi-DV models and the sum of the total energies of the (n−1)Li-G, (n−1)Li-SV, or (n−1)Li-DV models and a free Li atom. Atomic charges were calculated by the natural orbital analysis method (NBO 7.0, [42]). For the 6Li-G, 6Li-SV, and 6Li-DV models, the total partial densities of unoccupied C 2p states of 40, 41, or 42 central C atoms and the sum of Li 2s and 2p states of 6 Li atoms were plotted. The calculated intensities were broadened by Lorentz functions with a full width at the half maximum of 0.4 eV and normalized per one considered atom.

## 3. Results and Discussion

### 3.1. Characterization of As-Grown PC

**Figure 2:**
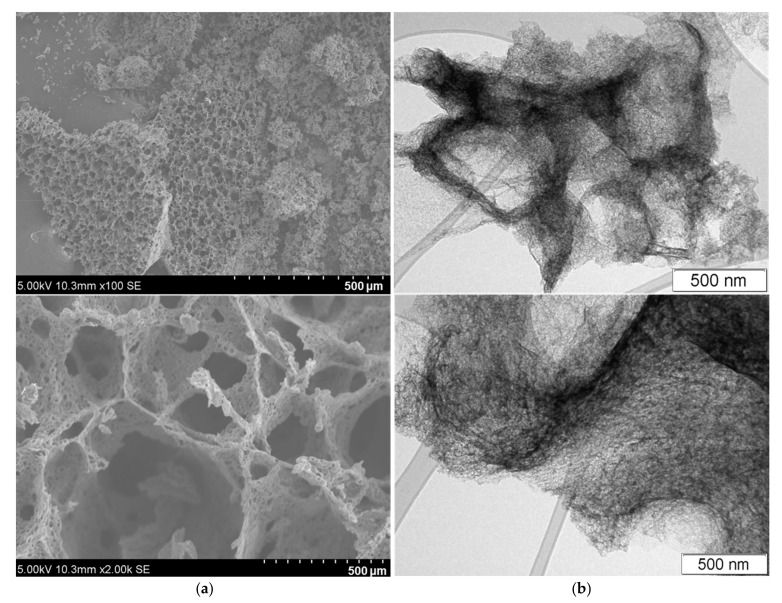
SEM (**a**) and TEM (**b**) images of PC.

SEM images demonstrate the spongy 3D architecture of the PC sample, in which carbon walls with a thickness of less than 1 μm separate interconnected micropores (Figure 2a). TEM images reveal that the primary carbon walls have a secondary nanoporous structure: disordered sp^2^ carbon thin layers surround quasi-spherical nanopores. According to our previous low-temperature nitrogen adsorption study of PC, it has a specific surface area of 931 m^2^ g^−1^ and a pore volume of 3.3 cm^3^ g^−1^, while the pore size varies from 2 to 20 nm [23].

**Figure 3:**
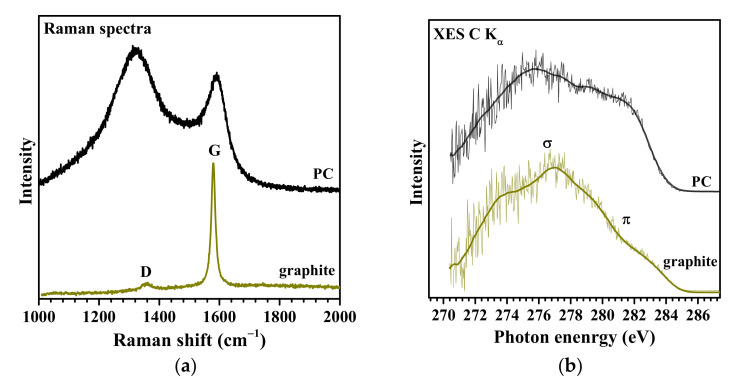
Raman scattering (**a**) and XES C K_α_ (**b**) spectra of PC and graphite.

The defectiveness of PC is evaluated by Raman scattering and XES spectroscopy. Figure 3a,b show the Raman and XES C K_α_ spectra of PC in comparison to the spectra of natural graphite. The Raman scattering spectrum of graphite is represented by two typical bands: a narrow G band at 1580 cm^−1^ associated with symmetric stretching vibrations of carbon–carbon bonds and a defect-induced low-intensity D band at 1360 cm^−1^ [43] (Figure 3a). The Raman spectrum of PC also contains G and D bands, which indicates that the walls have a local structure similar to graphite [44] and consist of sp^2^-hybridized carbon atoms in aromatic cycles linked to a 3D framework. The broadening of both bands and the high intensity of the D band are due to low atomic ordering in the graphene layers and a large number of structural defects in the PC sample.

The XES C K_α_ spectrum is formed as a result of the filling of vacancies created on the C 1s core levels with electrons from the C 2p valence states. According to the one-electron model, such a transition is accompanied by the emission of photons with an energy approximately equal to the energy difference between the levels involved in the transition. The XES C K_α_ spectrum of graphite exhibits a high-intensity feature at 277.0 eV and a shoulder at 282.0 eV associated with σ- and π-type molecular orbitals, respectively [45] (Figure 3b). The C K_α_ spectrum of PC demonstrates features characteristic of graphite, which confirms the sp^2^ hybridization of carbon atoms. However, the intensity of the π peak in the PC spectrum is significantly higher than that of graphite, which may be due to the presence of dangling bonds at the edge C atoms. Quantum chemical modeling showed that the intensity of the π maximum increases with a decrease in the size of graphene fragments or with an increase in the number of vacancy defects [46].

### 3.2. Effect of Lithium Deposition

**Figure 4:**
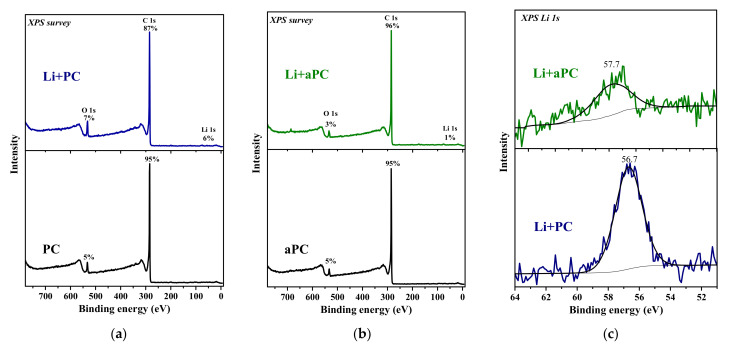
Survey XPS spectra of PC (**a**) and aPC (**b**) before and after Li deposition (Li + PC and Li + aPC). XPS Li 1s spectra of Li + PC and Li + aPC (**c**).

Survey XPS spectra detect carbon and about 5 at% oxygen on the surface of PC and aPC (Figure 4a,b). After the deposition of Li, the Li 1s line appears in the spectra of both samples. The surface concentration of Li for PC is 6 at%, which is six times higher than for aPC. This means that Li is predominantly fixed on the surface of PC but penetrates deeper into the volume of aPC. The XPS Li 1s spectra of lithiated samples are described by a single component at a binding energy (BE) of 57–58 eV (Figure 4c), close to that for LiC_6_ intercalated graphite (57.0 eV) [47] and for thermally deposited lithium on natural graphite (56.6 eV) [48]. It should be noted that the components from metallic Li at 55 eV and lithium oxide at 56 eV [49,50] do not significantly contribute to the spectra of lithiated samples.

**Figure 5:**
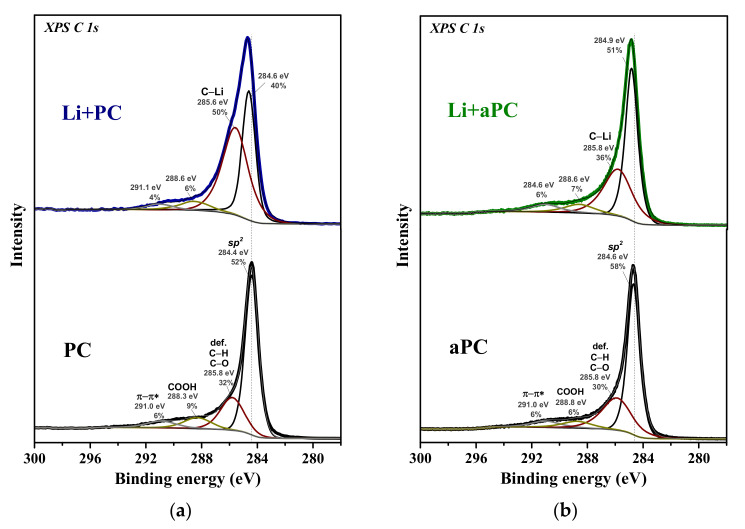
XPS C 1s spectra of PC (**a**) and aPC (**b**) before (bottom spectra) and after deposition of Li (upper spectra, Li + PC and Li + aPC).

The XPS C 1s spectra of PC and aPC before and after Li deposition are fitted by four components centered around 284.5, 285.8, 288.5, and 291.0 eV (Figure 5). The first and most intense component has a BE close to that of graphite and refers to sp^2^-hybridized carbon atoms [51]. The component at 285.8 eV can be assigned both to carbon atoms chemically modified with oxygen or hydrogen and to structural defect states [52,53]. The components at high BEs correspond to carboxyl groups (288.3–288.8 eV) and the π–π* shake-up transition (291.0 eV). For both PC and aPC samples, the oxygen concentration does not exceed 5 at%, although the contribution of the components at 285.8 eV and 288.3–288.8 eV is very large to the total area of the C 1s spectra. Therefore, it can be assumed that structural defects make the main contribution to the component at 285.8 eV. The width, intensity, and BE of all components in the C 1s spectrum of aPC do not differ practically from those for PC with the exception of a slight decrease in the intensity of the component at 285.8 eV. The XPS method does not reveal any significant changes in the composition and structure of PC that occurred as a result of annealing. The Li deposition causes the sp^2^ component to shift by 0.2 eV towards higher BE values and the component at 286.0 eV to become more intense (Figure 5a,b). Previously, DFT modeling of a partially lithiated single-walled carbon nanotube revealed that the shift of the main sp^2^ component is associated with nonlithiated areas and is the result of a shift in the Fermi level due to electron doping, and C atoms located near adsorbed Li give rise to a component at ~285.8 eV (denoted as C–Li for lithiated samples) [36]. The intensity of this component increases from 32 to 50% for PC and from 30 to 36% for aPC as a result of Li deposition. The higher the intensity of the C–Li component, the higher the surface concentration of Li.

**Figure 6:**
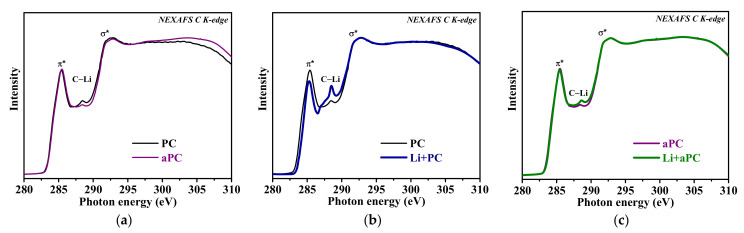
NEXAFS C K-edge spectra of PC and aPC (**a**), and PC (**b**), and aPC (**c**) before and after deposition of Li (Li + PC and Li + aPC).

Figure 6 compares the NEXAFS C K-edge spectra of PC and aPC before and after lithium deposition. The two resonances at 285.4 and 292.5 eV are typical for graphite and reflect the antibonding π* (C=C) and σ* (C=C) states [54]. The broad and smooth shape of π* and σ* resonances as well as the presence of a weak peak at 288.3 eV indicate an excess of structural defects and an admixture of oxygen- or hydrogen-containing surface groups [55]. The shape of the π* and σ* resonances does not change after PC annealing, but the feature between them somewhat decreases due to the atomic rearrangement or the removal of defects upon annealing (Figure 6a). In the spectra of both samples, the deposition of Li results in a reduction in the intensity of the π* resonance and the appearance of a new peak at 288.5 eV (denoted as C–Li in Figure 6b,c). However, these changes are more pronounced in the spectrum of PC than of aPC. Only a few works report the Li-induced changes in the NEXAFS C K-edge spectra of graphites [48,56,57]. The decrease in the intensity of the pre-peak region of the π* resonance is associated with the fact that Li valence electrons occupy the bottom of the conduction band in graphite. For highly oriented pyrolytic graphite crystals, a similar new peak appeared in the NEXAFS spectra in the region between π* and σ* resonances as a result of thermal lithiation or electrochemical intercalation of Li to the composition of LiC_6_, and the authors explained this peak as attributable to shifted σ* (C=C) states [56,57]. The shift in σ* states to the low-energy region was associated with a change in the C=C bond lengths and, consequently, the electronic band structure of graphite due to electron doping from Li; in this case, the shift in π* (C=C) states was not detected. The emerging spectral feature between the π* and σ* resonances is difficult to explain by the ionic nature of the lithium–carbon bonding. Earlier in our work, the peak at 288.5 eV in the NEXAFS C K-edge spectrum of thermally lithiated natural graphite was attributed to the formation of covalent σ bonds between Li and C atoms located at the edges of graphite flakes [48]. 

The carbon–lithium bonding in lithium–graphene systems is not purely ionic but has a significant contribution from the covalent interaction. In hydrogenated graphene, the H atoms are located on top of the C atoms, and due to the covalent bond formed, the C atoms become sp^3^ hybridized. Unlike H, the chemical bonding of graphene with Li atoms does not induce the transformation of carbon orbitals from sp^2^ to sp^3^ hybridization. In LiC_6_, Li atoms are located above the centers of carbon hexagons. Similar to H, Li has one valence electron and donates electron density from the 2s orbital to the lowest unoccupied π* states of graphite. However, quantum chemical calculations revealed the role of the empty Li 2p states in the dative bonding with carbon [29]. Li has a sp^2^ hybridization state, exhibits a π acceptor character, and accepts an excess electronic density from the 2p_z_ orbitals of carbon. Such additional dative bonds can explain the nature of multiple Li–C bond paths and the atomic arrangement in Li–graphene systems. Thus, checking the density of unoccupied states and the structure of molecular orbitals of lithiated defective graphene allows one to understand the experimental findings in the NEXAFS data.

It can be assumed that structural defects in graphene layers play a key role in the bonding between carbon and lithium and the nature of the Li adsorption process. The absence of carbon atoms in the lattice (vacancy defects) is the most common type of structural defect in graphitic materials [58]. DFT calculations showed that vacancy defects in graphene effectively increase the binding energy of metal and lead to more uniform distribution of metal over the surface of graphene [59]. A strong bond between Li and C atoms on vacancy defects can be responsible for blocking defects and preventing the intercalation of Li into the space between graphitic layers through defects [60]. Only the presence of large vacancy defects in graphene can provide a high Li storage capacity [61].

### 3.3. DFT Modeling

**Figure 7:**
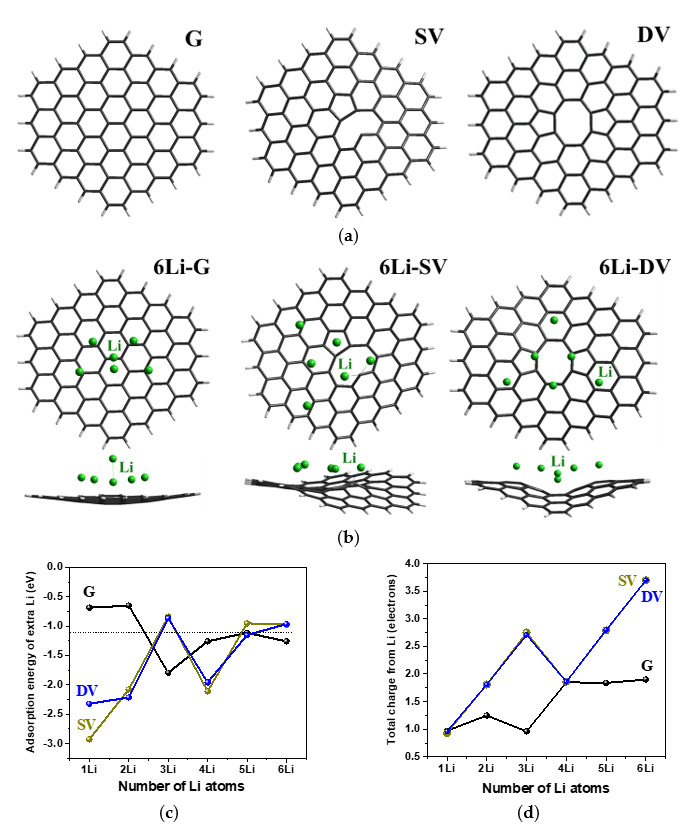
Optimized structures of graphene fragment (G) and fragments with single vacancy (SV) and diatomic vacancy (DV) (**a**). Optimized G, SV, and DV models with six lithium atoms (6Li-G, 6Li-SV, 6Li-DV) (**b**). Adsorption energies for newly added Li atoms (**c**) and charge transfer from all Li atoms (**d**) in nLi-G, nLi-SV, and nLi-DV models, where n varies from 1 to 6.

To reveal chemical bonding and changes in the electronic structure of lithiated defective carbon materials, DFT calculations of Li adsorption on a defect-free graphite fragment (G) and two graphene fragments with single vacancy (SV) and diatomic vacancy (DV) in the central part of the graphene fragment G were carried out (Figure 7a). We only considered the optimal size of the graphene fragment with a calculated C–C bond length of 0.142 nm. The distance from a single Li atom to the nearest C atom in model G is 0.235 nm, and the Li adsorption energy is −0.7 eV, which is in good agreement with previous reports [28]. As the size of the carbon model increases, the value of the Li adsorption energy can increase [30,62,63], but when the number of C atoms is several tens, the fragment size has very little effect on the local structure and adsorption energy of the Li atom. In the lithiated models, the number of Li atoms increased gradually until the last added Li ceased to interact with graphene. The total number of Li atoms was six for G, eight for SV, and seven for DV. Taking into account only one layer of Li atoms closest to the graphene plane, the limit stoichiometry is LiC_8.4_ for 5Li-G, LiC_5.8_ for 6Li-SV, and LiC_6.7_ for 7Li-DV. This result correlates well with previously published calculation data for Li and Na adsorption on graphenes, where it was shown that more dense metal monolayers are formed on the defective graphene than on defect-free graphene [64]. The optimized structures of the models with six Li atoms (6Li-G, 6Li-SV, 6Li-DV) are shown in Figure 7b. Multiple interactions between each Li atom and the nearest C atoms are found. The shortest Li–Li separation (0.31 nm for 6Li-G, 0.28 nm for 6Li-SV, and 0.30 nm for 6Li-DV) is larger than what was found in diatomic lithium, indicating a lack of Li–Li bond paths. For 6Li-G, the shortest Li–C bond is 0.25 nm, which is only 0.03 nm longer than that in LiC_6_. The presence of defects in the graphene structure results in a decrease in the Li–C bond length to 0.21–0.24 nm for 6Li-SV and 0.22–0.25 nm for 6Li-DV.

The dependencies of the adsorption energy for each last Li atom and the total charge transferred to graphene on the number of the Li atoms are shown in Figure 7c,d, respectively. The adsorption energy of a single Li atom is –0.7 eV for G, –2.9 eV for SV, and –2.3 eV for DV, which is consistent with previously published calculations [65,66]. Adsorption of the first two Li atoms at SV and DV sites is energetically more favorable than the adsorption on the defect-free graphene (Figure 7c). However, for the following Li adatoms, the adsorption energy is in the range from −2 to −1 eV and varies approximately equally for all structures. As the number of Li adatoms increases from one to six, the value of electron doping increases from 1 to 1.9e for G and to 3.9e for SV and DV (Figure 7d). Structural defects in graphene promote shorter C–Li distances and higher charge acceptance due to the weaker delocalization of π electrons and corrugation of defective graphene.

**Figure 8:**
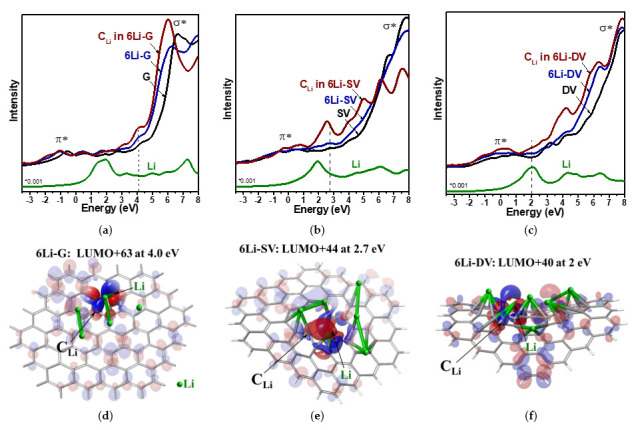
Partial density of unoccupied C 2p states for 42 central C atoms in G and 6Li-G (**a**), 41 central C atoms in SV and 6Li-SV (**b**), 40 central C atoms in DV and 6Li-DV (**c**), partial density of unoccupied Li 2s + 2p states in lithiated models (curve Li) and unoccupied C 2p states of the lithiated C_Li_ atoms denoted by arrows in 6Li-G (**a**,**d**), 6Li-SV (**b**,**e**), and 6Li-DV (**c**,**f**). Unoccupied molecular orbitals, which demonstrate large C_Li_ 2p_z_ orbitals towards the lithium–carbon bond path (**d**–**f**). Colors represent the different phases of the wave function.

Figure 8a–c show the partial densities of unoccupied states for 2p orbitals of C atoms located in the central part of graphene models with and without six Li atoms. The intensity of the curves is normalized per atom. The formation of C=C bonds gives π* states at energies below 5 eV and σ* states at energies above 5 eV. For all models, the addition of Li atoms causes not only the shift of π* and σ* states to lower energies due to the doping but also the appearance of new peaks between these states. At the same energy, the density of Li 2s and 2p states has a maximum (curve “Li” in Figure 8a–c). Therefore, they can overlap with graphene π* states, thus increasing the density of C 2p_z_ states.

The molecular orbital analysis of unoccupied states in lithiated models reveals the hybridization of Li 2s and 2p orbitals and the bonding σ-type interaction between π* orbitals of graphene and sp^2^ hybridized orbitals of Li (denoted as C π*–Li sp^2^). The π* system of graphene may participate in the concurrent acceptance of Li 2s electron density and the back-donation of extra charge to empty Li sp^2^ orbitals. Li interacts with graphene not only via the ionic interaction but also through the formation of a covalent bond of the donor–acceptor type. Such a dative character of chemical interaction results in the formation of additional σ-type bonding and increases the order of the C–Li bonds. In all lithiated graphene fragments, the orbitals related to the C π*–Li sp^2^ interaction are found. They have energies ranging from 0 to 6 eV and are located on the C atoms nearest to Li (C_Li_). As an example, the pronounced C π*–Li sp^2^ interactions are detected in unoccupied molecular orbitals: the 63^rd^ orbital above the lowest unoccupied molecular orbital (LUMO + 63) at 4 eV for 6Li-G, LUMO + 44 at 2.7 eV for 6Li-SV, and LUMO + 40 at 2 eV for 6Li-DV (Figure 8d–f). These Mos consist of sp^2^ orbitals of one or more Li atoms and π* orbitals of graphene. For the 2p_z_ orbital of a C_Li_ atom located near Li, increased overlapping with Li orbitals is observed. Partial densities of unoccupied C 2p states for C_Li_ atoms show well-defined peaks in the energy range from 0 to 4 eV, which are absent for fragments without Li (Figure 8a–c). The C π*–Li sp^2^ interactions are stronger in lithiated defective graphene than in defect-free graphene. Shorter C–Li distances and a denser arrangement of Li atoms can explain this result. Thus, the quantum chemical modeling shows that graphene fragments enriched with structural defect regions adsorb more Li atoms, have stronger C–Li interaction, and have higher electron doping than defect-free graphene. This result explains the experimental XPS and NEXAFS data.

### 3.4. Electrochemical Testing

**Figure 9:**
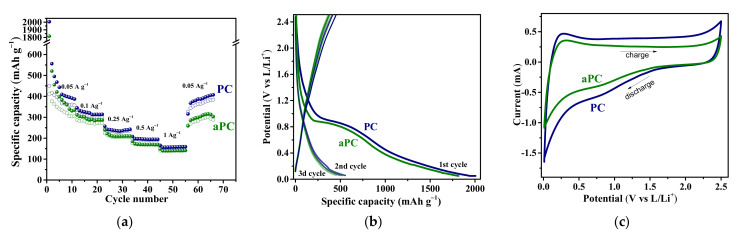
Electrochemical performance of PC and aPC anodes in LIBs: gravimetric capacity dependence on current density and cycle number (**a**), charge–discharge curves for the first three cycles at a current density of 0.05 A g^–1^ (**b**), and CV curves at a potential scan rate of 0.5 mV s^−1^ (**c**).

Figure 9 shows the results of electrochemical testing of PC and aPC in LIBs. The PC electrode has a higher capacity (411–164 mAh g^−1^ at a current density of 0.05–1 Ag^−1^) than the aPC electrode (303–140 mAh g^−1^ at 0.05–1 Ag^−1^). The capacity of PC at the first discharge cycle is ~2000 mAh g^−1^ and decreases to ~560 mAh g^−1^ at the first charging cycle. The aPC electrode demonstrates lower corresponding values of ~1800 and ~525 mAh g^−1^. A large drop in the initial capacity observed for PC and aPC is commonly associated with the formation of a passivating solid electrolyte interphase (SEI) layer [67]. The drop increases with increasing surface area and the number of defects and functional groups [68]. In the next cycles, the PC electrode has good stability with a capacity retention of 90–99%. In contrast to PC, the aPC electrode contains fewer defects, which leads to a greater decrease in the capacity over the entire cycling range.

At 0.05 Ag^−1^, the capacity of both PC and aPC is comparable to the theoretical capacity of graphite (Figure 9a). In graphite, the intercalation of Li^+^ ions into the interlayer space occurs at potentials of about 0.1 V, which manifests itself as a plateau in the charge–discharge curves [69]. However, unlike graphite, the discharge–charge curves of porous carbon electrodes demonstrate an extended sloping region in the voltage range of 0–1.2 V, which makes the main contribution to the capacity (Figure 9b). The low-voltage plateau corresponding to the intercalation of Li into graphite structures is weakly expressed. The electrochemical behavior associated with the surface adsorption of Li^+^ was found to be similar for various carbon nanomaterials [26]. There is the formation of a double electric layer and, as a consequence, the polarization of the carbon electrode. The adsorption of Li^+^ occurs in a wide range of potentials and depends on the presence of functional groups and defects on the surface. This process dominates for lithium and sodium storage in PC materials.

The CV curves of PC and aPC electrodes have a quasi-rectangular shape, which indicates the presence of adsorption and desorption processes at the interface between electrode and electrolyte and the formation of double electric layers (Figure 9c). In addition, the CV curves exhibit weak redox peaks at 0.1 V vs. Li/Li^+^ from lithium intercalation and at 0.7 V from lithium chemisorption on defects. Thus, the higher capacity of the PC electrode is associated with the higher adsorption of Li^+^ on defects. Annealing reduces the number of defects and, consequently, the amount of stored Li. It should be noted that the defective carbon surface also provides a high loss of Li at the first discharge cycle due to its strong binding to defects, resulting in high irreversible capacity. Chemical prelithiation of anode material, for example, by immersion into lithium-containing solutions, can be used to decrease the first irreversible capacity loss and improve the electrode stability stability.

**Figure 10:**
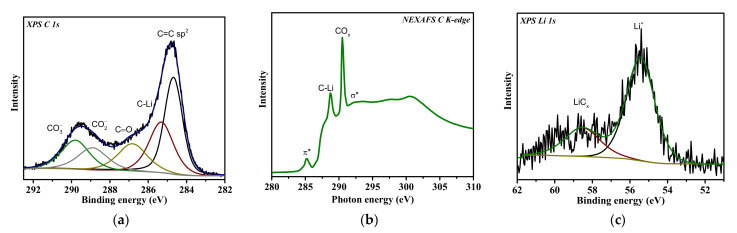
XPS C 1s (**a**), NEXAFS C K-edge (**b**), and XPS Li 1s (**c**) spectra of electrochemically lithiated PC electrode after extraction from the cell discharged to 0.01 V vs. Li/Li^+^.

The electrochemically lithiated PC electrode extracted from the cell after the 65th cycle at the final potential of 0.01 V vs. Li/Li^+^ was examined by XPS and NEXAFS spectroscopy. The survey XPS spectrum revealed 54 at% carbon, 17 at% oxygen, 24 at% lithium, and less than 1 at% impurity elements. Oxygen is mainly present on the electrode surface in the composition of lithium-containing carbonates (Li_2_CO_3_, LiHCO_3_, and Li_2_C_3_H_6_O_3_), which form the fully lithiated SEI layer [70]. The Li/C ratio on the electrode surface is 1.4, which is more than 0.3–0.7 for carbonates, indicating the presence of Li in the composition of the carbon electrode. The XPS C 1s spectrum of the sample revealed a set of components corresponding to sp^2^ carbon at 284.7 eV and carbon bonded with oxygen from the SEI layer at higher Bes of 286.9, 288.8, and 290.0 eV (Figure 10a). According to the XPS study of thermally lithiated samples, the component at 285.7 eV can be assigned to lithiated regions (C–Li) in the PC material. An intense peak at 290.5 eV in the NEXAFS C K-edge spectrum of the sample proves the presence of carbonate species (Figure 10b), while the decrease in the intensity of the π* resonance peak and the peak at 288.7 eV are most likely caused by the chemical interaction between π* (C=C) states and Li. The XPS Li 1s spectrum of electrochemically lithiated PC reveals at least two different chemical states of Li (Figure 10c). The lower BE peak centered at 55.5 eV is related to Li in carbonates, while the higher BE component at 58 eV can be assigned to Li interacting with C in the PC electrode.

## 4. Conclusions

In summary, in situ XPS and NEXAFS experiments on the adsorption of thermally evaporated Li on as-grown PC and PC annealed at 400 °C (aPC sample) show that the concentration of adsorbed Li on the surface of the former nanomaterial is 6 at% and higher than that of the latter one (1 at%). Structural defects are responsible for the high adsorption of lithium on porous carbon nanomaterials and their improved electrochemical capacity in LIBs. The NEXAFS spectroscopy study of unoccupied electronic states of carbon in the environment of adsorbed Li reveals a new peak at an energy of about 288.5 eV, the relative intensity of which is higher for the defective PC sample. According to our DFT calculations, this peak is due to dative chemical bonding between the π* (C=C) orbitals and the sp^2^ hybridized Li orbitals. Defects in the graphene lattice lead to a greater C π*–Li sp^2^ overlap, stronger Li–C interaction, and higher electron transfer from adsorbed Li to the C substrate. NEXAFS spectroscopy probing of the chemistry between alkali metals and sp^2^ carbon can be applied for adsorption, electrochemical energy storage, and sensing applications.

## Data Availability

Not applicable.
